# Management Strategy in Non-Limb-Threatening Acute Ischaemia of Limbs: Should We Rethink?

**DOI:** 10.1155/2016/8146295

**Published:** 2016-10-24

**Authors:** Syed M. Asim Hussain, Thomas Joseph

**Affiliations:** North Cumbria University Hospitals NHS Trust, Cumberland Infirmary, Newtown Road, Carlisle, UK

## Abstract

The Society of Vascular Surgery and the International Society of Cardiovascular Surgery identify three types of acute limb ischaemia to inform prognosis and management. Type 1 limb ischaemia is non-limb-threatening and is currently managed conservatively. We describe three cases of Type 1 limb ischaemia with femoropopliteal occlusion that were managed differently. The first case was initially managed conservatively but resulted in an adverse outcome following worsening of ischaemia. Overall, the cases managed with earlier intervention had good outcomes suggesting that conservative management alone may not be sufficient despite resolution of symptoms. The trend in other vessel diseases such as NSTEMI and TIA is towards earlier intervention, for example, PCI and CEA. It is likely that acute limb ischaemia has a similar natural history to these conditions. It is time to consider earlier revascularisation in selected patients with non-limb-threatening ischaemia.

## 1. Introduction

Acute limb ischaemia (ALI) is a sudden reduction in limb perfusion. A trend of increasing admissions was noted with this common vascular emergency [1]. The Society for Vascular Surgery and the International Society of Cardiovascular Surgery provided a clinical classification to aid its management [2]. This involves clinical assessment to determine viability of the limb and conservative management when it is not threatened. This is similar to the approach used in managing Transient Ischaemic Attacks (TIA) and Non-ST Elevation Myocardial Infarction (NSTEMI) in the past. We discuss a few cases of ALI that raise caution with this approach.

## 2. Case  1

A 45-year-old lady with history of claudication, diabetes, and smoking was admitted on developing right foot pain suddenly. The pedal pulses were not palpable but had Doppler signals. The leg was viable with no neurological deficit or muscle tenderness indicating Type 1 acute ischaemia. She was on aspirin and atorvastatin. She received anticoagulation in the acute phase and a CT angiogram (CTA) showed short occlusion of distal superficial femoral artery (SFA) with patent popliteal artery and two-vessel run-off in the calf. She started mobilising well. Vascular MDT recommended right SFA angioplasty after 6 weeks for fear of embolisation in the immediate period. However she was readmitted in 4 weeks with worsening pain and blue toes.

An angiogram on second admission showed occlusion of the right SFA and run-off vessels. She underwent SFA thrombectomy and on-table angioplasty of popliteal artery but no run-off vessels were demonstrated. Subsequently, she underwent exploration of foot vessels and fem-pedal bypass but this also occluded leading to below-knee amputation.

## 3. Case  2

A 73-year-old lady presented with right foot and calf pain of 2-week duration. Initially, her toes were cyanosed with foot being cold, pale, and numb. She smoked and had hypertension. The popliteal, posterior tibial and dorsalis pedis pulses could not be palpated. Subsequently, her symptoms improved but she still had short distance claudication suggesting Type 1 ischaemia.

CTA confirmed distal SFA stenosis and occlusion in the popliteal artery with two-vessel run-off. She was treated with stent graft resulting in having palpable pedal pulses and resolution of symptoms.

## 4. Case  3

A 43-year-old man presented with sudden onset of rest pain. His right foot was pale and cold with sudden onset of paraesthesia. He smoked and had claudication. No pulse was palpable below the femoral level on the right side. He was treated with anticoagulation resulting in improvement of symptoms indicating Type 1 ischaemia of a non-limb-threatening nature. He mobilised well on the ward but reported his work being affected by claudication for a couple of weeks prior to admission. His Ankle Brachial Pressure Index (ABPI) was 0.5.

CTA showed right popliteal artery occlusion proximally with patent calf vessels. He underwent right popliteal thrombectomy but this reoccluded despite anticoagulation. All his symptoms except paraesthesia resolved following a femoropopliteal bypass graft.

## 5. Discussion

The Society for Vascular Surgery and the International Society of Cardiovascular Surgery provided a clinical classification for assessment of acutely ischaemic limbs. They identified three categories of ischaemia. In Type 1 ischaemia, the limb is not under threat and, in Type 3 ischaemia, the limb is nonviable.

In acute limb ischaemia clinical signs of muscle tenderness and neurological deficit are used to identify acutely threatened limbs or Type 2 ischaemia, where urgent revascularisation is recommended. Type 1 ischaemia is defined as the sudden onset of symptoms with no residual calf tenderness or neurological deficit.

The first patient had acute-on-chronic limb ischaemia. She deteriorated rapidly while waiting for deferred angioplasty. On initial assessment, she was deemed to have Type 1 (non-limb-threatening) ischaemia. Therefore, she was managed in accordance with the current recommendation for managing Type 1 ischaemia with the best medical therapy alone. However, this resulted in worsening of symptoms and subsequent limb loss.

In the second case, a primary stent graft was used as her symptoms were present for a week and therefore thought to be ALI with stabilisation of the thrombus. The stent graft was used primarily to exclude any unstable thrombus that could potentially cause distal embolisation.

In the third case, thrombectomy failed in the immediate postoperative period despite ensuring good inflow during the surgery and continuing anticoagulation postoperatively. The reocclusion may indicate the unstable nature of the endothelium.

All three cases were categorised to be Type 1 ischaemia with no limb-threatening features such as numbness, muscle tenderness, or inaudible Doppler signals. All patients received anticoagulation which is standard practice in our unit. Since chronicity was suspected in the third case with short distance claudication, an Ankle Brachial Pressure Index test was performed which was 0.5 which could have been Rutherford stage 4 as per the classification for chronic ischaemia. However, the acute onset of symptoms suggested an acute ischaemic problem rather than chronic ischaemia in this case.

Current clinical practice does not mandate urgent revascularisation for patients presenting with Type 1 ischaemia because these are not considered to be limb-threatening situations. Urgent interventions to improve the circulation are only recommended in Type 2 ischaemia when features of continuing tissue damage such as muscle tenderness or neurological dysfunction are present. This may be a cost-effective strategy if progression of non-limb-threatening ischaemia to limb-threatening ischaemia is a rare event.

Current practice is similar to the approach taken in managing acute presentations such as unstable angina, Non-ST Elevation Myocardial Infarction (NSTEMI) and Transient Ischaemic Attacks (TIA) in the past. However, in the case of unstable angina and NSTEMI, the management has become more proactive with earlier invasive intervention in high risk groups [3–7]. A similar change has been seen in the management of TIA due to severe carotid disease [8, 9]. The clinical group of non-limb-threatening acute ischaemia (Type 1) may represent a similar heterogeneous group of unstable patients. The cases discussed above support this view although more evidence needs to be sought. A more rigorous approach to expedited revascularisation may help at least a subgroup of patients presenting with Type 1 ALI. The developments in cardiology and stroke medicine suggest this and it is very likely that the natural history of acute limb ischaemia is similar to these vascular conditions, despite resolution of symptoms.

Current practice in managing ALI recommends conservative management alone for patients with non-limb-threatening ischaemia and this may not be sufficient in all such cases. This group of patients can potentially deteriorate quickly and more observational data is necessary in this group of patients to determine if this is the right strategy.

## 6. Conclusion

This small case series suggests the possibility for further deterioration in Type 1 ALI when managed conservatively and such cases may benefit from proactive interventions. However, more research is needed to determine the optimal management strategy and characterise the patients likely to benefit from earlier intervention.
